# Identifying gaps in consumer health library collections: a retrospective review

**DOI:** 10.5195/jmla.2021.895

**Published:** 2021-10-01

**Authors:** Eleni Giannopoulos, Michelle Snow, Mollie Manley, Katie McEwan, Andrew Stechkevich, Meredith Elana Giuliani, Janet Papadakos

**Affiliations:** 1 eleni.giannopoulos@uhnresearch.ca, Research Analyst, Cancer Health Literacy Research Centre, Cancer Education, Princess Margaret Cancer Centre, Toronto, Ontario, Canada; 2 michelle.snow@uhn.ca, Librarian, Princess Margaret Cancer Centre, Toronto, Ontario, Canada; 3 mollie.manley@gmail.com, Princess Margaret Cancer Centre, Toronto, Ontario, Canada; 4 highlandkate@sympatico.ca, Princess Margaret Cancer Centre, Toronto, Ontario, Canada; 5 andrew.stechkevich@uhnresearch.ca, Cancer Health Literacy Research Centre, Cancer Education, Princess Margaret Cancer Centre, Toronto, Ontario, Canada; 6 meredith.giuliani@rmp.uhn.on.ca, Radiation Oncologist, Medical Director Cancer Education, Princess Margaret Cancer Centre, Toronto, Ontario, Canada; 7 janet.papadakos@uhnresearch.ca, Codirector, Cancer Health Literacy Research Centre, Cancer Education, Princess Margaret Cancer Centre; Provincial Head, Patient Education Cancer Care Ontario; Assistant Professor, University of Toronto, Toronto, Ontario, Canada

**Keywords:** neoplasm, consumer health, gap analysis, collection development

## Abstract

**Background:**

The objective of this study was to determine if search request forms, which are used when a patron's request for information cannot be fulfilled at the time of contact with the library team, can be used to identify gaps in consumer health library collections.

**Case Presentation:**

Search request forms were collected from 2013 to 2020 and analyzed independently by two reviewers. Search request forms were included if they were complete and contained a record of how the request was fulfilled. Descriptive statistics were used to summarize patron characteristics. Search request forms were iteratively coded to identify themes in the data and determine if resources provided to patrons could be found within the library collection. The study team subsequently reviewed search request forms to determine reasons for identified gaps. Two hundred and forty-nine search request forms were analyzed. Six main content themes were identified: 1) understanding the cancer diagnosis, 2) cancer treatments, 3) understanding disease prognosis, 4) support during and after treatment, 5) natural health products and therapeutic effects in oncology, and 6) research literature. The majority of patrons were patients (53%). Over half (60%) of the submitted search request forms reflected collection gaps, and many (16%) contained queries for information about rare cancer diagnoses. The main reason that queries could not be satisfied was that there was limited consumer health information on the requested topics (53%).

**Conclusions:**

Search request forms are a useful resource for assessing gaps in consumer health library collections.

## BACKGROUND

Cancer patients and their families want to be well informed about their diagnosis throughout their cancer journey, regardless of prognosis [1, 2]. Provision of information has a wide range of benefits including improved understanding about disease, treatment, and methods of management [3–6]. Meeting cancer patients' informational needs can lead to lowered anxiety, improved coping, and overall health [7]. As cancer care continues to advance, consumer health libraries are challenged to consistently update and expand their collections to respond to the changing information needs of patient and family patrons [8, 9].

A growing body of evidence underscores the value of consumer health libraries. Consumer health libraries positively impact patient health and save time for health care providers who are readily able to access information about important topics such as side effect management, specific drugs, and clinical guidelines [10, 11]. Knowing more about their cancer and treatment options allows patients to exert greater control over their health [12], and patients who receive more information about treatment options are more active in health-related decision-making [13]. The value of health libraries is further acknowledged when we consider that information on the Internet is not regulated, and health information of varying relevance, quality, currency, and readability levels is widely accessible on the Internet to patients and families [14]. This can present a challenge for individuals who are unable to assess the credibility of online information or who may be subject to “information overload” [15]. This might be of particular importance to patients and families with low health literacy, for whom self-navigating through the Internet and locating reliable health information can be a significant challenge [14]. Consumer health libraries can intervene by providing individualized support to patients and families who seek information about cancer-related topics. In addition, consumer health libraries are consistently credited for providing more than just improved patient understanding or knowledge; they offer unique, individualized social support to cancer patients who may feel anxious, especially at the time of their diagnosis or prior to the start of treatment [16]. Furthermore, consumer health libraries can have a tremendous impact on patient safety and satisfaction [17].

Search request forms are specialized tools that contain several open- and closed-ended questions and are administered by a librarian to solicit detailed information about patrons' requests for information. The librarian completes the search for resources, and the search yield is emailed, sent by post, or picked up by the patron in person.

Here, we explored whether an archive of search request forms could be used to identify gaps in a library collection as an adjunct to more traditional collection development approaches. Our specific aims were to determine if search request forms could be used to 1) identify gaps in a library collection and 2) understand why these gaps exist.

## CASE PRESENTATION

### Search request forms

The consumer health library in Princess Margaret Cancer Centre, a large, urban and academic cancer center in Canada, has an extensive collection of vetted, high-quality cancer-related resources [3, 18]. These resources are available in several different media formats including pamphlets, books, e-books, videos, and curated lists of reliable websites. A librarian is responsible for collection development and reviewing online and print resources to ensure that resources are able to address the needs of patrons. It is common protocol for the librarian to provide an information consult to locate resources within both the physical and virtual library collections for patient and family patrons [3]. Information consults are quick reference interviews to determine an individual's information needs, which is especially relevant considering that some information needs are explicit while some are implicit and may be more difficult for patrons to articulate [3]. In the event that the request for information cannot be fulfilled immediately because the information is not available in the library collection (e.g., research articles), the patron is constrained by time and cannot wait, or the query is complex and requires a further search online, a request for information can be made using a search request form.

An archive of search request forms collected from 2013 to 2020 was examined and independently reviewed by two reviewers. All collected search request forms were included if the section asking for “type of information requested” was complete and the form contained a record of how the request for information was fulfilled. The librarian attaches a print summary of the search yield to each completed search request form. The summary is formatted as a letter addressed to the patron and includes references and hyperlinks to the materials provided. Search request forms were excluded if the information requests were not related to cancer (e.g., information about ulcerative colitis) or were incomplete (e.g., fewer than three fields completed).

### Data analysis

In order to analyze the contents of search request forms, data from each eligible form were entered into an Excel database [19]. Data included information about the patron (e.g., patient or family), cancer diagnosis, preferred method for receiving information (e.g., email), and purpose of the request (e.g., treatment-related, diagnosis). Once the data from the forms were entered, each search request was coded to document whether the request was a single request for information (e.g., seeking treatment-related information) or contained more than one request (e.g., seeking information about primary diagnosis, potential treatment options, and strategies for side effect management).

#### Aim 1. Identification of collection gaps

A study team member iteratively coded each search request form to explore themes in the requests using an inductive approach [20, 21]. For example, several search request forms included requests for basic information about uncommon cancers and were coded as “rare cancers.” A second study team member reviewed the themes and discussed opportunities to refine them with two study team members until consensus was reached. A third study team member (i.e., the librarian) was consulted if any discrepancies or questions arose. Following thematic coding, study team members reviewed the search yield of each search request form to determine if the resources sent to the patron could be found within the physical or virtual library collection or if the request required a further search. Requests that could not be fulfilled by resources available in the library collection were deemed to be gaps.

#### Aim 2. Identification of reasons for collection gaps

Study team members reviewed themes from the search request forms to determine reasons for the identified gaps. Study team members discussed whether 1) there was limited consumer health information on the topic, 2) the information could not be accessed due to paywall restrictions or controlled membership, 3) the query was uncommon, or 4) there was abundant consumer health information but it did not meet health literacy best practices. Information that follows health literacy best practices is accurate, easy to understand, actionable, and accessible [22]. Limited consumer health information indicated topics for which there was limited information published, and controlled membership referred to resources that required subscriptions in order to access the information.

### Library patron characteristics

Between 2013 and 2020, 260 search request forms were submitted to the library. Duplicate requests were removed, and the remaining search request forms were reviewed against inclusion and exclusion criteria. Two hundred and forty-nine search request forms met inclusion criteria and were analyzed. The majority of patrons were patients (Table 1). The most commonly reported cancer diagnosis was breast cancer, followed by hematologic (e.g., central follicular lymphoma, marginal zone lymphoma) and gynecologic (e.g., ovarian cancer, cervical cancer) cancers. Most patrons indicated that they preferred information from the search request to be delivered via email. The majority of search requests contained multiple requests for information.

**Table 1:** Search request form requester information (n=249)

**Patron information**		**n (%)**
Patron	Patient	132 (62.9%)
	Family member	56 (22.5%)
	Other (e.g., staff, friend, visitor)	23 (9.2%)
	Unspecified	38 (15.3%)
**Cancer diagnosis**		
Cancer type	Breast	58 (23.3%)
	Hematology	41 (16.5%)
	Gynecology	25 (10.0%)
	Gastrointestinal	23 (9.2%)
	Genitourinary	22 (8.8%)
	Sarcoma	21 (8.4%)
	Head and neck	15 (6.0%)
	Unspecified	12 (4.8%)
	Lung	11 (4.4%)
	Other (e.g., neuroendocrine cancer)	9 (3.6%)
	Melanoma	7 (2.8%)
	Brain	5 (2.0%)
**Information delivery**		
Method of delivery*	Email	173 (69.5%)
	Pick-up	61 (27.7%)
	Mail	19 (9.2%)
	Unspecified	10 (4.0%)
Type of request
	Multiple requests for information	162 (65.1%)
	Single request for information	87 (34.9%)

* Some respondents selected more than one option

### Content categories in search requests

Six main content themes were identified in the search request forms: 1) understanding the cancer diagnosis, 2) cancer treatments, 3) understanding disease prognosis, 4) support during and after treatment, 5) natural health products and therapeutic effects in oncology, and 6) research literature. The majority of search requests asked for information to help in understanding the cancer diagnosis (39%), cancer treatments (31%), and the cancer prognosis (13%). Fewer search requests asked for research literature (8%), information about support during and after treatment (5%) and natural health products and therapeutic effects (4%).

Queries regarding the cancer diagnosis included requests for information about cancer type (e.g., staging, pathology, and genetic factors), information about diagnostic testing and safety (e.g., magnetic resonance imaging (MRI) or computed tomography scanning), and multilingual health information about the cancer type (e.g., information in Chinese or Italian).

Treatment-related requests included queries for specific chemotherapy regimens (e.g., carboplatin), radiotherapies (e.g., proton therapy), targeted or hormonal therapies (e.g., tamoxifen), and immunotherapies (e.g., Revlimid). Additional treatment requests regarded side effects and their management (e.g., pain or fatigue) and treatment decisions (e.g., lumpectomy versus mastectomy).

### Resources provided to library patrons

**Figure 1:**
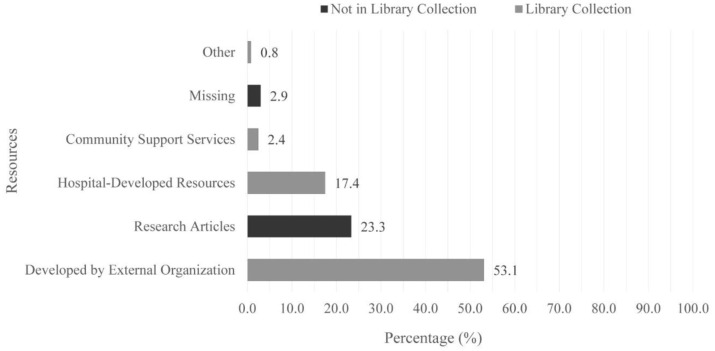
Resources provided to library patrons.

The librarian consulted several library resources (i.e., a combination of resources developed by external organizations as well as by the hospital) as well as research information to fulfill patrons' requests. The majority of sources provided to library patrons were found within the library collection and were developed by external organizations (i.e., Canadian Cancer Society, American Cancer Society, National Cancer Institute, and Mayo Clinic) [17]. Hospital-developed resources were also provided to patrons. External and hospital-developed resources included webpages, books, pamphlets, and videos. Additional resources consulted included published research articles (Figure 1).

### Identifying and explaining collection gaps

Over half of the submitted search request forms reflected gaps in the library collection (n=150, 60%) (Table 2). Of these, the majority contained queries for information about rare cancer diagnoses (e.g., leiomyosarcoma and myelodysplastic syndrome) (17%). Regarding cancer treatments, most search requests contained queries for information on reconstructive surgery (4%), followed by treatment decisions related to breast reconstructive surgery (e.g., saline vs. silicone implants) (3%). The top queries related to understanding the cancer prognosis were about treatment-related survival and quality of life (5%) and chances of metastasis (5%). Regarding support during and after treatment, most search requests asked for information about support groups (e.g., locations and types of groups) (3%). Most queries for information about natural health products and therapeutic effects in oncology were about medical cannabis (4%).

**Table 2:** Content gaps identified in search requests

Content gap categories	Content gap subcategories	Frequency	Reasons for gaps			
			*Limited consumer health information*	*Access controlled membership*	*Uncommon query*	*Abundant consumer health information that does not meet health literacy best practices*
**Understanding the cancer diagnosis**						
**Learning about cancer type**	Rare cancer diagnosis	25	X			
	Pathology (e.g., growth, morphology)	2	X		X	
	Environmental risks (e.g., radiation exposure)	5	X		X	
**Diagnostic testing and safety**	Visual inspection with acetic acid for cervical cancer	1	X		X	
	Gadolinium-based contrast agents for MRI	2	X		X	
**Cancer treatments**						
**Radiotherapy**	Integrated boost radiation	1	X			
	Targeted intraoperative therapy	1	X			
	Metabolically adaptive radiation	1	X			
**Surgery**	Reconstructive surgery (e.g., breast and cervical cancer)	6	X			
	Amputation (e.g., preventing sarcoma metastasis)	1				X
**Side effects of treatment**	Dental issues (e.g., tooth sensitivity)	1	X			
	Vision loss	1	X			
	Gynecologic problems (e.g., vaginal discharge)	1	X			
	Axillary web syndrome	1	X			
**Treatment decisions: breast cancer**	Tamoxifen vs. aromatase inhibitors (e.g., Letrozole)	4	X			
	Tamoxifen vs. radiotherapy	1	X			
	Lumpectomy vs. mastectomy	3	X			
	Breast reconstructive surgery; saline vs. silicone	5	X			
	Cyclophosphamide vs. docetaxel chemotherapy	1	X			
	Hormonal therapy vs. radiotherapy	1	X			
**Treatment decisions: ovarian cancer**	Radical hysterectomy vs. fertility-saving treatment	2	X			
	Surgery vs. chemotherapy	1	X			
**Treatment decisions: lung cancer**	Lobectomy vs. segmentectomy or pneumonectomy	1	X			
**Treatment decisions: brain cancer**	Temodar/radiation vs. PCV/radiation	1	X			
**Treatment decisions: kidney cancer**	Partial vs. radical nephrectomy	1	X			
**Access to clinical trials**	Drug manufacturers	1	X			
**Understanding disease prognosis**						
**Survival statistics and evidence**	Chances and signs of recurrence	5	X			
	Age-related survival	3				X
	Treatment-related survival and quality-of-life	8				X
	Risk of developing secondary conditions (e.g., diabetes due to prednisone)	1				X
**Metastasis**	Chances of metastasis	8				X
	Secondary cancers	3				X
**Support during and after treatment**						
**Support resources**	Support groups (e.g., locations, types of groups)	5			X	
	Podcasts	1	X			
	Inpatient programs	1			X	
**Health and wellness**	Memory improvement strategies	1	X			
**Natural health products and therapeutic effects in oncology**						
**Antioxidants and drug interactions**	Anti-angiogenesis (e.g., turmeric, flax)	1	X	X		
**Therapeutic effects and access**	Ganoderma (i.e., fungal medicine)	1	X	X		
	Cannabis (e.g., pancreatic cancer)	6	X	X		
	Red reishi mushroom	1	X	X		
	Vitamin C	2	X	X		
	Thermal therapy	1	X	X		
	Mistletoe treatment	2	X	X		
	Oxygen therapy	1	X	X		
**Queries for latest research evidence from the literature**						
**Effectiveness of chemotherapy drugs for breast cancer**	Paclitaxel	1		X		
	Capecitabine	2		X		
	Doxorubicin	1		X		
	Aromatase inhibitors	1		X		
	Cyclophosphamide	1		X		
	Docetaxel	1		X		
**Effectiveness of hormonal/targeted therapies for breast cancer**	Stivarga	1		X		
	Arimidex	1		X		
	Letrozole	1		X		
	Tamoxifen	1		X		
**Effectiveness of immunotherapy for breast cancer**	Revlimid	1		X		
**Effectiveness of surgery for breast cancer**	Auxiliary lymph node dissection	2		X		
**Surgery for prostate cancer**	Prostatectomy	1		X		
**Radiotherapy**	Exposure (e.g., breast cancer)	1		X		
	Health risks (e.g., prostate cancer)	1		X		
**Therapeutic evidence of natural health products**	Cannabis	2		X		
**Cancer types**	Myelodysplastic syndrome and myelofibrosis (polycythemia vera)	2		X		
	Malignant mixed Mullerian tumor	1		X		
	Leiomyosarcoma	1		X		
	Adenocarcinoma	1		X		
	Stomach cancer (e.g., H. pylori)	1		X		
	Ovarian cancer	1		X		
	Anal dysplasia disease	1		X		
	Anaplastic astrocytoma (e.g., hormone effects)	1		X		

The main reason queries could not be satisfied at the time of the information consult was there was limited consumer health information on the requested topics (53%). This was the case for all topics related to understanding the cancer diagnosis, cancer treatments, and natural health products and therapeutic effects in oncology. A large proportion of queries contained topics that were not accessible due to paywall or membership restrictions (25%), such as information about natural health products and therapeutic effects in oncology. For a small portion of queries, there was abundant consumer health information available that did not meet health literacy best practices (14%) or the query was uncommon (9%). This was the case for most queries related to understanding the disease prognosis. For example, one patron asked for information about lung cancer metastasis, including growth rates and patterns of spreading. Resources provided to satisfy this request included webpages and findings from the American Society of Clinical Oncology (ASCO), which consists of clinical guidelines, research, and some educational materials for patients and families [23, 24]. However, ASCO information regarding lung cancer metastases was intended for clinician use and, as such, did not meet health literacy best practices.

## DISCUSSION

Our findings revealed that the majority of requests were submitted by cancer patients, and the most commonly requested topics were about breast cancer, with specific queries for information about advanced topics related to diagnosis, treatment, and prognosis. While the current collection contains a vast amount of information on specific types of cancer and treatments, this finding is testimony to patrons' complex information-seeking behaviors and the evolving nature of cancer and novel treatments [5, 25, 26].

Librarians use a multitude of approaches to evaluating their collections and identifying gaps. Although traditional collection evaluation tools, such as interlibrary loan statistics or library cataloging [27], continue to serve as important tools for collection development and gap identification, some new collection evaluation tools have begun to emerge, including demand-driven acquisition [28]. Demand-driven acquisition allows users to recommend titles directly to librarians and is facilitated by some digital library lending services like OverDrive [28]. A less technical example of demand-driven acquisition described by Leonard et al. [29] was to develop and expand a print collection to include resources for patients and families. The authors found that patients and families increasingly used the print collection, and new items were added based on consumer requests. However, with the transition to digital technology and the integration of electronic materials and acquisition of external databases in e-libraries, collection development practices continue to evolve [30, 31].

Search request forms can be added to the repertoire of collection evaluation tools, as they can be used as tools to respond to patrons' information needs and to identify gaps in library collections. Search request forms serve as a conduit for direct patient feedback by eliciting details about patients' specific needs for information and, more broadly, provide a clearer understanding about the needs of the patient population on a larger scale. Through this service, librarians are able to quantify gaps that are identified and analyze why these gaps exist. Furthermore, search requests can be monitored over time to determine whether the query reflects a persistent and long-term need or a need that is emergent based on advances in medicine.

The literature consistently shows breast cancer patients to be avid information seekers, with information about disease and treatment being the most sought-after topics [32, 33]. Our findings complement the literature in that we found that the majority of search requests were submitted by breast cancer patients, whose queries mainly regarded cancer diagnosis, treatments, and prognostic information [34]. One of the most significant gaps identified in our study was information pertaining to treatment decisions among this population. As the decision-making preferences of breast cancer patients evolve throughout the course of their treatment and cancer journey [35, 36], one way to respond to this need and address this gap may be to engage clinician subject matter experts to develop resources that present the advantages and disadvantages of common treatment options for various breast cancer diagnoses.

We found that while the majority of resources provided to patrons were from the library's collection (74%), 23% of materials provided to patrons were published research articles, and 8% of all search requests contained queries that specifically requested information from the research literature. This finding supports previous findings that as more time passes after a cancer diagnosis, patients can become more interested in the most up-to-date or current research [37]. However, this information may be difficult for patients to access due to paywalls and membership restrictions. With the increasing number of open access journals, access to this information may be less of an issue in the future. Additionally, a number of external websites were consulted to respond to patrons' queries. One study by Fulda et al. found that websites used by hospital and library websites were reliable and contributed to the library's expanding collection [38].

Furthermore, advancements in naturopathic medicine and the use of natural health products as an adjunct to conventional medicine in oncology has led to a greater demand for patient education resources. As such, libraries may wish to consider subscribing to, or purchasing licenses for, centralized digital natural health databases such as the Natural Medicines database [39] as a strategy for addressing patrons' needs for this information [40].

Not surprisingly, a significant gap identified in our study was a lack of resources on rare cancers, such as sarcomas. Patients and families dealing with rare cancers often experience a number of issues including alienation, anxiety, and worry due to fears that health care providers have limited understanding about their disease, lack of information about appropriate treatment, and limited evidence to help with decision-making processes [41]. This may in turn negatively impact communication between patients and their doctors [42], which may leave patients feeling unsupported [43]. However, the challenge in responding to requests about rare cancers lies in the fact that there is a dearth of literature and consumer health information on this topic [42]. As such, cancer centers may need to develop these patient education materials in-house to fill this gap.

This finding points to an opportunity for librarians and patient educators to connect with clinical teams and patient education programs at other cancer centers and hospitals to determine whether they have begun to develop resources to address the same gaps. Leveraging existing resources will not only allow for timely provision of information and closing of existing collection gaps but could reduce duplicated efforts across centers and costs for patient education resource development. Our previous study reports that cancer centers spend significant amounts of time and money on patient education material development and that patient education programs could reduce some of these costs if they share resources developed by one another [44, 45]. As such, connecting with patient education programs and libraries in other cancer centers may help librarians quickly address collection gaps.

In a small proportion of gaps, we identified that consumer health information did not meet health literacy best practices. This was the case for cancer prognostic information, including survival statistics, metastasis, and quality of life. Although cancer patients and families actively seek information on survival statistics and disease progression [46, 47], the vast majority of patient education materials have reading grade levels well above the recommended targets [48–50]. Furthermore, cancer patient education materials frequently fall below the acceptable standards for understandability and actionability [48, 50]. This can have significant implications for cancer patients [51], including confusion, inability to be involved in treatment decisions, issues related to goal planning and prioritization, or decline in self-management behaviors [52, 53]. One strategy to address this gap may be for consumer health librarians to act as advocates for health-literate consumer health practices and provide feedback to organizations to raise awareness. Additionally, plain language assessments can be done using a number of different validated tools, including readability calculators [54, 55] and the Patient Education Materials Assessment Tool [56], which may be used to evaluate understandability and actionability of resources.

This study employed a retrospective review of data. In the context of cancer, advances in medical treatments happen at a quick pace and, as such, a limitation of this study is the issue of temporality, as a request made in 2013 may not be relevant in 2020.

In conclusion, search request forms are a useful resource to explore gaps in consumer health libraries collections. Specifically, search request forms can be used to identify content gaps and develop an understanding of why the gaps exist, which can inform future collection development initiatives.

## Data Availability

Data associated with this article cannot be made publicly available because they contain personally identifiable information. Access to data can be requested from the corresponding author at janet.papadakos@uhnresearch.ca and may be subject to Institutional Review Board restrictions.
